# Growth Performance, Carcass and Meat Quality Traits of Three Rabbit Lines Under Heat Stress Conditions

**DOI:** 10.3390/ani15213200

**Published:** 2025-11-03

**Authors:** Emanuele Pontalti, Zsolt Matics, Marco Cullere, Zsolt Szendrő, Zsolt Gerencsér, Bianca Palumbo, Antonella Dalle Zotte

**Affiliations:** 1 Department of Animal Medicine, Production and Health, University of Padova, Viale dell’Università 16, 35020 Legnaro, Padova, Italy; emanuele.pontalti@phd.unipd.it (E.P.); bianca.palumbo@phd.unipd.it (B.P.); antonella.dallezotte@unipd.it (A.D.Z.); 2 Department of Animal Sciences, Széchenyi István University, H-9200 Mosonmagyaróvár, Hungary; matics.zsolt@ga.sze.hu; 3 Institute of Physiology and Nutrition, Hungarian University of Agriculture and Life Sciences, Kaposvár Campus, Guba Sándor Str. 40, 7400 Kaposvár, Hungary; szendro47@gmail.com; 4 Institute of Animal Sciences, Hungarian University of Agriculture and Life Sciences, Kaposvár Campus, Guba Sándor Str. 40, 7400 Kaposvár, Hungary; gerencser.zsolt@uni-mate.hu

**Keywords:** rabbit genotypes, high temperature, heat stress, slaughter traits, meat quality, fatty acids, proximate composition, oxidation, sensory analysis

## Abstract

**Simple Summary:**

Globally, climate change is resulting in more frequent and intense heat waves in different areas of the world, which represent a challenge for the livestock sector. Rabbits, whose production plays an important role both in subsistence farming and in industrial farming, are particularly sensitive to heat stress (HS). In fact, temperatures above 27–28 °C are sufficient to cause impaired welfare, depressed behavior and production losses. Until now, research has focused on common commercial hybrids, whereas limited information is available for other commercial lines, which is a key gap to be addressed. The present research studied how three different rabbit lines of the Pannon Rabbit Breeding Programme (Hungary) responded to chronic HS in terms of productive responses, meat quality characteristics and sensory features. The aim was to identify the most suitable line to be farmed in challenging environmental conditions: Pannon Large—PL vs. Pannon White—PW vs. Pannon Ka—PK. The results highlighted that PK and PW rabbits were the most impacted by chronic HS, while the better results displayed by the PL line suggest its potential in mitigating HS-related negative effects. However, the current paucity of scientific information regarding this line suggests the need for further investigation into tailored management practices, aimed at enhancing line performance and traits under challenging environmental conditions.

**Abstract:**

Given the yearly challenging environmental scenario with more and more frequent and intense heat waves, the livestock sector has to find affordable and sustainable solutions to face the expected increase in meat demand by 2050. Among livestock species, rabbits are particularly sensitive to heat stress (HS) but, paradoxically, the scientific background on the response of different genetics to environmental stressors like HS is rather scarce. This is a significant gap, especially considering that most of the demographic growth, and meat demand, is expected in developing countries where rabbits play a key role in subsistence farming. Therefore, this research investigated the effects of environmental temperature (Control—20 °C; High—28 °C) on growth performance, slaughter traits and meat quality of three Hungarian rabbit genotypes (Pannon Large—PL; Pannon White—PW; Pannon Ka—PK). Animals (n = 360) were housed in wire-mesh cages (3 animals/cage) in two separate controlled-temperature rooms (60 rabbits/genotype/room), from 5 to 11 weeks of age, during which they received ad libitum feed and water. Even if the three genotypes were exposed to the same environmental challenge, they exhibited different responses. The PL line showed superior performance, with the highest carcass weight and yield (*p* < 0.001), and the greatest water-holding capacity (*p* < 0.01) in the loin muscle. The PW rabbits showed the largest reduction in overall weight gain (−24.7%; *p* < 0.001) and the lowest decrease in feed conversion ratio (−3.20%; *p* < 0.001). PK rabbits experienced the greatest reduction in total dissectible fat (−34.6%; *p* < 0.001) and hind leg lipid content (−20.3%; *p* < 0.01), with the highest proportion of polyunsaturated fatty acids (*p* < 0.01), which fostered meat lipid oxidation (*p* < 0.05). As expected, these differences in performance and meat quality traits reflected the distinct selection criteria and genetic background of these genotypes: the PL is a paternal line, the PK is a maternal line, and the PW is a productive line. Regarding the temperature effect, PK and PW genotypes were the most impacted by chronic HS: PW rabbits suffered the largest performance depression, while PK rabbits showed the worst carcass and meat quality traits. Instead, PL rabbits demonstrated the best outcomes under chronic HS, showing the greatest productive efficiency and satisfactory meat quality traits.

## 1. Introduction

Globally, the average temperature rises each year, leading to more frequent and intense heat waves, particularly in tropical and Mediterranean regions, but also impacting many continental countries [1]. The livestock sector currently faces challenges in meeting the growing demand for animal-based food products. Accordingly, affordable and sustainable solutions will soon be pivotal to address the expected 70% increase in demand by 2050 [2]. The latter will mainly involve developing countries, where rabbits still play an important role in subsistence farming. As a homoeothermic animal with a thick fur covering its entire body and a limited number of functional sweat glands [3], rabbits possess a notably limited thermoneutral zone (15–21 °C) [4], which makes them highly susceptible to heat stress (HS). Above the upper temperature limit, rabbits’ homeostasis is disrupted, resulting in impaired welfare, depressed behavior and, consequently, significant production losses [5]. So far, the effects of HS have been acknowledged by the scientific community in different livestock species, including poultry [6,7], pigs [8], ruminants [9,10] and rabbits as well [11,12,13]. Concerning the latter species, the majority of literature focuses on assessing the impact of HS on common commercial hybrids and the New Zealand White, with limited information available for other commercial lines.

Over the past three decades, the Kaposvár University (Hungary) has been conducting a rabbit breeding program (Pannon Rabbit Breeding Programme) focused on creating different meat-producing rabbit lines with distinct selection goals [14]. Since 1988, the program has obtained three distinct rabbit lines: the Pannon Ka (PK—maternal line), the Pannon White (PW—productive line) and the Pannon Large (PL—paternal line). Initially, the PW and PL lines were pre-selected for their average daily gain, even though, in the PW line, this selection criterion was then replaced by the litter weight at 21 d of age. A significant milestone of this selection program was the innovative integration of computer tomography (CT), a key technological implementation that allowed the non-invasive measurement and selection of longissimus and thigh muscles volume in PW and PL lines over several decades and a few years, respectively, to improve the slaughter values. As a side-effect of the selection carried out on PW rabbits to increase the tight muscle volume, the weight of fat depots also decreased [15]. Despite their importance in Hungarian rabbit meat production, the scientific background on these lines, especially regarding their response to different environmental stressors like HS, is rather scarce. Available studies are indeed limited to individual lines, including the PK [16,17,18] and PW [19], while data on the PL line’s response are still missing. To address this gap, the present study, an extended version of Matics et al. [20], subjected these three rabbit genotypes to chronic HS conditions to investigate their productive responses, meat quality characteristics and sensory features, aiming to identify the most suitable line to be farmed in challenging environmental conditions.

## 2. Materials and Methods

### 2.1. Animals, Experimental Design and Farming Conditions

The present trial was conducted at the experimental rabbit farm of the Hungarian University of Agriculture and Life Sciences (Hungary) under the approval of the Institutional Animal Welfare Committee (MATE KC MÁB/5-5/2021) as the animal welfare body of the University. Rabbits were handled following the principles stated in the Commission Directive 2010/63/EU of 22 September 2010 on the protection of animals used for scientific purposes.

A total of 360 weaned rabbits belonging to three different genotypes, namely Pannon Ka (PK), Pannon White (PW), and Pannon Large (PL), were randomly allocated into two separate rooms (n = 60 rabbits/genotype/room) from weaning (5 week—wk) to slaughter (11 wk; 6 wk in total). The two rooms differed only in average environmental temperature, which was set at 20 °C in the control room and at 28 °C in the heat stress room. Environmental temperature was strictly controlled using an air conditioning system (Fujitsu Air conditioning system, ARYG30LMLE, Fujitsu General Limited, Suenaga, Takatsu-ku, Kawasaki, Japan) and were continuously measured during the experiment with an EBI 300 USB data collector (ebro Electronic GmbH, Ingolstadt, Germany). Overall, the average environmental temperatures were 19.7 ± 1.4 °C and 28.7 ± 1.6 °C in the controlled and HS rooms, respectively. Relative humidity (RH) was also recorded throughout the experimental period: the recorder values were 68.0 ± 8.9% and 52.4 ± 7.2% in the controlled and HS rooms, respectively. Temperature and RH were then used to compute the THI—temperature–humidity index [20], which resulted in 19.2 (no HS) and 26.6 (very severe HS) THI for controlled and HS rooms, respectively. Rabbits were housed in wire-mesh cages (57 × 38 × 30 cm; 3 rabbits/cage) and fed with two commercial pelleted diets according to their age (5–9 wk: digestible energy—DE: 9.94 MJ/kg, crude protein—CP: 15.7%, crude fibre—CF: 19%, with medication; 9–11 wk: DE: 10.6 MJ/kg, CP: 16.3%, CF: 17.7%, without medication), while water was available from nipple drinkers. Both feed and water were provided ad libitum throughout the experimental period. A lighting schedule of 16 light: 8 dark was applied in both rooms throughout the entire period.

### 2.2. Performance Data, Slaughtering and Carcass Traits

During the trial, individual live body weight (BW) and cage feed intake (FI) were measured at weeks 5, 7, 9 and 11, while mortality was checked daily. Afterwards, individual daily weight gain (DWG) and feed conversion ratio (FCR) were calculated on a cage basis.

At 11 wk of age, 240 rabbits (2 rabbits/cage, 40 rabbits/genotype/room) were randomly selected, fasted for 6 h, then transported to a professional slaughterhouse located 200 km from the rabbit farm (transport duration: 4 h). Upon arrival, rabbits were individually weighed to determine the slaughter weight, electrically stunned and then slaughtered by severing the carotid arteries and jugular veins. After being exsanguinated and skinned, 15 carcasses/genotype/room (n = 90) were randomly chosen for data collection and later dissected according to the World Rabbit Science Association recommendations [21]. Following the same procedures, the blood, skin, and the entire gastrointestinal tract of the selected carcasses were collected and weighed to calculate their incidence on the slaughter weight. Carcasses were then stored in a ventilated room at 4 °C for 24 h to register the chilled carcass (CC) weight and its drip loss the following day. Thereafter, the weight of liver, kidney, head, and reference carcass (RC) was measured, following the recommendations mentioned above. From the carcass, perirenal and scapular fat depots were removed, weighed and their sum yielded the total dissectible fat. All carcass parts, as well as both perirenal and scapular fat depots, were computed based on the CC weight. Each carcass was then divided into three portions (fore, mid, and hind part) by cutting between the 7th and 8th thoracic vertebrae and the 6th and 7th lumbar vertebrae. Lastly, the hind legs (HL) and *longissimus thoracis et lumborum* (LTL) muscles of each carcass were dissected and weighed to determine their yield based on the RC weight.

After carcass traits collection, all 90 carcasses were transported in chilled conditions to the Department of Animal Medicine, Production and Health (MAPS) of the University of Padova (Italy) for meat quality analyses.

### 2.3. Meat Physicochemical Properties

Upon arrival, all the left HL and LTL muscles were individually vacuum-sealed (99% vacuum level) inside polyethylene bags (CSV-41n ORVED machine, ORVED, Musile di Piave, Italy) and frozen at −40 °C for subsequent proximate and sensory analyses, respectively. Within 24 h post mortem, ultimate pH (pHu) and CIE L* (lightness) a* (redness) b* (yellowness) color values of both right HL and LTL were measured, specifically at the level of the *biceps femoris* (BF) muscle and the 5th lumbar vertebra, respectively [22]. Muscles’ pHu was read using a FE20 pH-meter (Mettler Toledo, Greifensee, Switzerland) equipped with temperature probe and calibrated with pH 4.00 and 7.00 solutions, while L* a* b* color coordinates were determined with a RM200QC colorimeter (X-Rite, Co., Neu Isenburg, Germany. Measuring Area: 8 mm; Measuring Geometrics: 45/0 Image Capture; Illuminant/Observer: D65/10), calibrated with a white plate. Both variables were measured in triplicate, and the average measurement was then calculated and used. Subsequently, both HL and LTL samples were vacuum-packed and frozen at −40 °C. After overnight thawing at 4 °C, all samples were removed from the bags, dried with paper towel and weighed to calculate the thawing losses. The thawed LTL muscles were vacuum-packed again and cooked for 20 min in a water bath set at 80 °C, until core temperature reached 74 °C. Once cooked, samples were cooled down to room temperature in iced water, extracted from the bags, dried with paper towel, and weighed again to calculate the cooking losses. The toughness of cooked LTL meat was determined based on Warner–Bratzler shear force, following the same methodology reported in Dalle Zotte et al. [23]. Separately, the thawed right HL were deboned to predict the carcass meat-to-bone ratio [24]. All bones were collected and weighed, while the meat was freeze-dried and, after 3 days, finely ground using a Retsch Grindomix GM 200 (Verder Scientific S.R.L., Pedrengo, Italy) mincer (4s at a revolution speed of 8500 rpm) to determine the fatty acid (FA) profile [23]. The HL femur was used to determine the Warner–Bratzler fracture toughness (expressed in Newtons) using a dynamometer Texture TA-HD (SMS—Stable Micro System, Godalming, UK) equipped with a 6 cm wide cell, and the load rate was 0.5 mm/s. The proximate composition was analyzed on freeze-dried left HL, following the methods of the Association of Official Analytical Chemists [25] to determine moisture (method n. 943.01), protein (method n. 2001.11) and ash (method n. 967.05) contents. Ether extract was analyzed after acid hydrolysis [26]. From the remaining meat, an aliquot of about 20 g was packed inside plastic bags and stored at −40 °C to determine the heme iron content [27], while another aliquot of 10 g was used for lipid oxidation [28] analysis. The latter was determined using a spectrophotometer (Hitachi U-2000; Hitachi, 209 Mannheim, Germany) set at 532 nm, measuring the absorbance of thiobarbituric acid reactive substances (TBARs). Lastly, the last portion of HL meat was used to determine the mineral profile [24], including the content of Ca (method n. 991.25), P (method n. 991.27), Na (method n. 956.01) and K (method n. 975.03). Minerals were determined through inductively coupled plasma optical emission spectrometry, performed with Spectro Arcos (SPECTRO Analytical Instruments, GmbH, Kleve, Germany) after microwave digestion (Milestone rotor set at 64-bar pressure).

### 2.4. Meat Lipids Extraction, Fatty Acid Profile Determination and Health Index Calculations

Rabbit meat lipids were extracted using modified accelerated solvent extraction (M-ASE) using a solvent mixture of chloroform/methanol 1:2 and in accordance with the method of Lee et al. [29]. After vacuum evaporation under a nitrogen stream, the fat content of meat samples was determined gravimetrically. Then, samples were trans methylated using a 4% H_2_SO_4_ methanolic solution, which allowed us to determine fatty acid (FA) methyl esters (FAMEs). In each sample, 0.5 mL of distilled water and 1.5 mL of *n*-heptane were added in order to obtain a biphasic separation. FAMEs were quantified through gas chromatography (Shimadzu GC17A, Shimadzu Italia, Milano, Italy), which was equipped with an Omegawax (Sigma-Aldrich Co. LLC, Saint Louis, MO, USA) 250 column (30 m × 0.25 μm × 0.25 μm) and flame ionization detector. The carrier gas was helium (constant flow of 0.8 mL/min). Both injector and detector temperatures were 260 °C. For identification, commercially available FAME mixtures (37-Component FAMEs Mix; Supelco Inc., Bellefonte, PA, USA) were used. Results were expressed as the % of total detected FAMEs. In addition, lipid health indexes (Atherogenicity—AI, Thrombogenicity—TI, Peroxidability—PI and hypocholestrolemic/Hypercholesterolemic—hH) were subsequently calculated as described by Mattioli et al. [30].

### 2.5. Meat Sensory Analysis

A ranking test was conducted on LTL meat samples after one month of storage at −40 °C. The panel was composed of 24 staff members of the MAPS Department, pre-screened for good health and accustomed to rabbit meat consumption. Panelists underwent four training sessions using fresh rabbit LTL meat to familiarize them with product quality traits. During training, they developed and standardized descriptors (toughness, juiciness, fibrousness, greasiness, and overall odor/flavor intensity). They also identified potential off-odors/flavors (pungent, cardboard, acid, metallic, livery, and rancid) according to the International Organization for Standardization [31]. The sensory analysis comprised n = 5 sessions, with n = 15 samples per treatment evaluated in individual booths with controlled conditions. Each panelist was provided with the necessary utensils, water, crackers, and paper towels. Samples were thawed (24 h at 4 °C), vacuum-sealed, coded, cooked (85 °C for 25 min) and served warm every day. Panelists received six samples (1/treatment) simultaneously and ranked them for each attribute from 1 (least intense) to 4 (most intense), also indicating the presence/absence of specific off-odors/flavors.

### 2.6. Statistical Analysis

Experimental data were processed following the general linear model (GLM) procedure in SAS 9.1.3 Statistical Analysis Software for Windows [32]. After conducting a Shapiro–Wilk test to evaluate normality, data were analyzed using a two-way analysis of variance (ANOVA) considering Genotype (G: PL vs. PW vs. PK) and Temperature (T: 20 vs. 28 °C) as fixed effects, as well as their interaction. Single animals were used as experimental units, except for the analysis of FI and FCR, where the experimental unit was the cage. Sensory analysis scores were evaluated through a mixed model (PROC MIXED), considering genotype and temperature as fixed effects, while panelists were set as the random effect. Least square means presented in tables were obtained and corrected using a Bonferroni test, considering significance at a 5% confidence level. A χ^2^ test was performed on the off-odor/flavor perception according to the Marascuilo procedure [33].

## 3. Results

### 3.1. Growth Performance

Rabbit growth performances were significantly influenced by genotype, temperature, and, in several cases, by their interaction (Table 1). As for the genotype effect, PL rabbits exhibited the highest BW, DWG, FI, and the best FCR (*p* < 0.001), whereas PK rabbits showed the lowest performance and the worst feed efficiency (*p* < 0.001). A high environmental temperature was shown to decrease the performance (*p* < 0.001) of all genotypes, including their BW (−14.3%), DWG (−20.7%) and FI (−25.6%), though the FCR decrease was a productive improvement (−7.00%; *p* < 0.001).

From the 7th wk of age, the three genotypes started to respond differently to chronic HS, as highlighted by the interaction between genotype and temperature (G × T). Indeed, HS led to a more pronounced reduction in the FI of PL and PW rabbits (−28.3% vs. −27.7%, respectively) compared to PK rabbits (−25.2%) in the period of 7–9 wk (*p* = 0.001). However, the greatest decline (*p* < 0.05) in growth rate (−29.3%) and BW (−14.8%) was observed exclusively in PW rabbits, while PL responded similarly to PK rabbits. Additionally, only PW rabbits exhibited an increased FCR (*p* < 0.01) under HS conditions (+2.60%), while PL and PK rabbits showed a comparable reduction (−7.00% and −6.70%, respectively). Considering the entire growth period, a G × T interaction was only observed in the DWG and FCR. Once again, the PW line emerged as the most susceptible to HS, displaying the largest reduction in DWG (−24.7%; *p* < 0.05) and the smallest improvement in FCR (−3.20%; *p* = 0.001). Conversely, PL and PK rabbits under HS had a similar response, with comparable reductions in both DWG (−18.6% vs. −18.7%, respectively) and FCR (−8.90% vs. −8.90%, respectively). Mortality data were recorded but not statistically analyzed and presented in tables, since only one PL rabbit housed at 20 °C and two PL rabbits housed at 28 °C died during the experiment.

### 3.2. Slaughter Results

Consistent with growth performances, the carcass traits of the rabbits were influenced by both genotype and environmental temperature and, for some traits, interactions were also observed (Table 2). At slaughter, PW carcasses showed intermediate weights, although they were the leanest (1.33% of total dissectible fat; *p* < 0.001) with the highest proportions in the hind part and HL (39.1 and 37.1%, respectively; *p* < 0.001). In contrast, PL rabbits were the heaviest (*p* < 0.001), consequently yielding the highest CC (*p* < 0.001) and RC (*p* < 0.001) weights. Nevertheless, the same genotype exhibited balanced resource allocation across the carcass, since its main cut proportions (i.e., fore, mid, and hind parts) were intermediate compared to the other genotypes (*p* < 0.05). Lastly, the PK genotype proved to be the least efficient among the three. It displayed the lightest CC and RC, along with the lowest yield in both hind part and HL (36.8 and 34.3%, respectively), as well as the largest fat depots (2.54%). Simultaneously, PK carcasses exhibited the highest proportions of liver, head and total dissectible fat (*p* < 0.001).

The HS condition decreased (*p* < 0.001) the CC (−13.4%) and RC (−13.2%) weight of all three genotypes, as well as the incidence of gastrointestinal tract (−11.1%), liver (−9.80%) and total dissectible fat (−29.2%; *p* < 0.001). Although the RC yield was not affected (*p* > 0.05), the hind part and HL proportions of all genotypes significantly increased (*p* < 0.001) under high temperature (+2.80%, and +4.30%, respectively). Essentially, only carcass fat proportions varied in function of the HS and the three genotypes, as highlighted by the G × T interactions for perirenal and total dissectible fat percentages (*p* < 0.05). Indeed, PK rabbits emerged as the most susceptible for these traits under HS conditions, showing the greatest losses in total dissectible fat (−34.6%; *p* = 0.04), followed by PL (−29.6%) and PW (−23.3%) rabbits.

### 3.3. Meat Physicochemical Properties

Results showed that the rabbit genotype did not affect the pHu and color characteristics of both BF (Table 3) and LTL (Table 4) muscles. Instead, PL rabbits showed the best water-holding properties in both their HL (*p* < 0.01) and LTL (*p* < 0.05) muscles, as well as the highest HL meat yield (*p* = 0.001) and the lowest LTL toughness (*p* < 0.001). Despite the LTL muscles of PW and PK lines displaying similar water-holding capacities, the meat of the PK line was notably tougher compared to the other lines. Conversely to genotype, environmental temperature had a significant effect on the muscle pHu and color traits of the LTL, increasing the pHu (+0.60%) value (*p* < 0.01) while reducing the yellowness (−6.40%; *p* < 0.05). Redness value was also reduced in the BF muscle (*p* < 0.05). Instead, HS did not affect the muscle water-holding capacities of both meat cuts (*p* > 0.05), except for the thawing loss of the LTL, which was reduced in the HS group (*p* < 0.05). A significant G × T interaction was observed only in the HL femur fracture toughness (*p* < 0.05). Indeed, HS markedly decreased femur toughness in PW (−14.6%) and, to a lower extent, in PK rabbits (−6.60%). In contrast, the greater resilience of PL rabbits to chronic HS was reflected in an improved femur toughness (+3.70%).

Examining the chemical composition of the HL meat (Table 5), PL rabbits exhibited the lowest protein (*p* < 0.01) content, while PK rabbits displayed the highest lipid content (*p* < 0.01) at the expense of water (*p* < 0.001), along with an increased mineral content (*p* < 0.01). Conversely, PW rabbits showed intermediate results but a higher oxidative status (*p* < 0.05) compared to the other genotypes. Chronic HS had a significant effect on the HL proximate composition (*p* < 0.01), decreasing heme iron content (−11.4%) while augmenting that of water (+1.30%). Similar to the trends observed at the carcass level, HS led to a reduction in meat lipid content, but to a different extent among genotypes. PK rabbits exhibited the highest lipid content decline (*p* < 0.05) in the HL meat (−20.3%), followed by PL (−11.6%) and PW rabbits (−10.1%).

Concerning lipids, the FA profile of HL meat was affected by both genotype and temperature (Table 6). Rabbits belonging to PL and PW genotypes displayed a similar FA profile, characterized by higher proportions of polyunsaturated FA (PUFA; *p* < 0.01) and total *n*-6 FA (*p* < 0.01) compared to PK rabbits. Furthermore, PW showed the highest proportion of alpha-linolenic acid (C18:3 *n*-3 *p* < 0.01) and consequently the greatest fraction of total *n*-3 FA (*p* < 0.01) among the genotypes. Also, PK rabbits’ HL meat exhibited the highest proportion of oleic acid (C18:1 *n*-9) and total monounsaturated FA (MUFA; *p* < 0.01). The observed changes in the FA profile of PL, PW and PK meat determined variations in the considered health indexes, which generally showed the worst outcomes in the meat of PK rabbits, with PL and PW displaying similar values. The HS condition decreased the total MUFA in meat (*p* < 0.01) and raised the meat linoleic acid (C18:2 *n*-6; *p* < 0.01) proportion, contributing to augmentthe total *n*-6 FA (*p* < 0.01) and thus overall PUFA (*p* < 0.01). This, together with the similar *n*-3 FA proportion in the meat of rabbits housed at 20 °C and 28 °C, increased the meat *n*-6/*n*-3 ratio (*p* < 0.01). Also in this case, relevant variations in the FA profile of HS meat compared to that of the Control group generated different health indexes: atherogenicity index (*p* < 0.05) improved in the meat of HS rabbits, whereas peroxidability (*p* < 0.01) and hypocholesterolemic/Hypercholesterolemic (*p* < 0.01) indexes worsened. Also, all three genotypes responded similarly under chronic HS conditions, as a G × T interaction was never observed.

### 3.4. Meat Sensory Traits

Perceived toughness was the sole sensory characteristic of the LTL meat that was affected by genotype (Table 7 and Table 8). Panelists consistently rated the LTL meat of PK rabbits as tougher compared to that of PW and PL rabbits, whose meat was identified as the most tender (*p* < 0.05). The HS condition was not a relevant factor when considering the other sensory traits of LTL meat.

## 4. Discussion

For the last thirty years, a genetic breeding program conducted at Kaposvár University, Hungary has led to the development of three rabbit lines with different characteristics, which currently play a major role in the Hungarian rabbit production [14]. Given the increasing frequency of HS events, there’s a growing need for more heat-tolerant genotypes across the entire livestock sector, including rabbits. Genetics offers one of the most valuable solutions to mitigate the negative effects of environmental stressors, prompting scientific research in obtaining commercial hybrids displaying balanced productive and resilience traits. Past scientific studies assessed the impact of HS on these rabbit lines selected by Kaposvár University [14,18,19], although these investigations were limited to individual lines. Moreover, until now, the response of the PL line to HS was unknown. Therefore, the present study aimed to compare these three rabbit genotypes to identify which one could show the highest adaptability under high environmental temperatures.

Given its higher adult BW compared to the other lines, the PL line expectedly showed the best growth performance among the three genotypes, followed by PW and PK rabbits. This was in line with previous studies [34,35], which concluded that the performances of a certain genotype were intimately correlated with its adult BW. Chronic HS negatively impacted the live performances of all three genotypes, with differing responses becoming significant from the 7th week of age. Over the entire growth period, PW rabbits were consistently the most impacted by chronic HS. This was observed despite their intermediate performance compared to the other genotypes which, in contrast, responded similarly under chronic HS. These findings differed to those of past studies, which indicated that commercial genotypes with higher overall performance were more impacted by HS compared to slow-growing or local breeds [36,37]. This observation was also reported in broiler chickens by Gogoi et al. [38], who attributed a diminished tolerance to HS in birds with a higher live BW, but of the same age and genotype. Interestingly, in the present study, the heaviest and best-performing genotype (PL line) was actually the most adaptable among the three. The higher susceptibility to HS of the PW line may stem from its limited body fat reserves. Indeed, under HS conditions, animals try to decrease their metabolic heat production to limit the increase in core body temperature. To achieve this, they change their metabolism from the use of thermogenic substrates (proteins and carbohydrates) to less thermogenic lipid breakdown. Thus, if lipid reserves are low, they may shift back to protein breakdown that produces more metabolic heat, making animals more sensitive to HS [39]. In PW rabbits, selective breeding for increased thigh muscle volume has led to reduced fat depots [15], thereby presumably limiting their capacity to mobilize energy under HS. This was supported by data in Table 2, which showed that the lowest fat reserves as well as the smallest reduction in fat under HS were found in PW rabbits, indicating a limited ability to mobilize energy that may have contributed to the observed results.

At slaughter, carcass weights reflected the rabbit live BW at 11 wk of age. Accordingly, PL rabbits exhibited the heaviest CC but the lowest yield, which was possibly due to a lower physiological maturity at slaughter [40]. PW rabbits showed intermediate carcass weights but higher yields in both CC and HL. This was a consequence of the long selection period for improving the volume of LTL and hind leg [14,41]. Furthermore, the lowest dissectible fat ratio found in PW rabbits was attributable to the selection for thigh muscle volume. This, as a side-effect, reduced the amount and ratio of fat depots [15]. As a maternal line, PK proved to be the least efficient genotype, yielding carcasses with the highest proportions of certain organs and dissectible fat, along with the lowest RC weight and yield. Essentially, both PL and PW carcasses were heavier and leaner than those of the PK line, denoting that CT-based selection had a relevant impact on carcass traits across genotypes. Under chronic HS, carcasses were lighter and leaner, as reported by other scientific studies [16,42]. However, the impact of HS varied across the genotypes in connection with the amount of fat depots, with the PK line exhibiting the greatest reduction in carcass adiposity. The contrasting responses of PW and PK rabbits to chronic HS suggested that the same environmental stressor elicited line-specific responses. Specifically, in PW rabbits, the HS condition primarily hampered the deposition of lean tissue, whereas in PK rabbits, the adverse effects of HS were more pronounced in adipose tissue. These findings are consistent with those of Matics et al. [17] who reported that, under chronic HS conditions, rabbits divergently selected for higher body fat content experienced greater fat loss compared to those selected for lower adiposity. This differential response may be attributed to the higher dietary energy requirements needed for fat deposition relative to lean tissue (muscle) development [43].

At a physical level, the genotype’s effect on the pHu and color traits of both BF and LTL muscles was negligible, as also observed by Dalle Zotte et al. [44]. In contrast, the CT-based selection improved the HL meat-to-bone ratio in both PL and PW lines, despite the differing water-holding capacities. PL rabbits showed the best outcomes, leading to a more tender LTL muscle compared to the other lines, a result also attributed to their lower maturity at slaughter [40]. Conversely, the PW line’s water capacities were similar to those of the PK line, which also had the lowest HL meat yield and the toughest LTL muscle. Chronic HS had a relatively mild effect on meat physical traits. It decreased the redness of BF muscles and the yellowness of LTL muscles while simultaneously increasing the pHu only in the latter muscle. This was likely attributable to the different muscle metabolism: the LTL muscle, being more glycolytic, relies more on glycogen stores compared to the BF muscle [45]. The depleted glycogen reserves resulting from the HS-induced reduction in FI could therefore have hampered the post mortem lactic acid production, resulting in higher meat pH values.

The past genetic selection resulted in PL and PW lines showing a similar HL chemical composition, with the exception of a lower protein content found in the first line, which might be due to the PL line’s shorter selection period compared to the PW one [14]. In contrast, the maternal PK line showed an HL meat richer in minerals and lipids, consistent with the enhanced fat depots found at carcass level. However, Dalle Zotte et al. [44] reported no differences in the proximate composition of both HL and LTL meat across these three genotypes. Separately, chronic HS decreased the meat heme iron and lipid content, the latter’s decrease being more pronounced in the HL of PK rabbits, consistently with the observations made at the carcass level.

Regarding lipids, the HL FA profile varied across genotypes. The CT-based selection increased the PUFA and total *n*-6 FA fractions, essentially due to higher linoleic acid proportions, which corroborated the findings of Dalle Zotte et al. [44]. Furthermore, the HL meat of PW rabbits exhibited the greatest *n*-3 FA fraction. This increase also made meat more susceptible to oxidation, as evidenced by the higher TBAR content compared to the other lines. In contrast, the PK line showed a lower PUFA fraction and, consequently, a greater MUFA proportion. Accordingly, the PL line had the most favorable lipid profile, presenting a high fraction of PUFAs (mainly *n*-6 FA) and intermediate MUFA proportions, which resulted in the lowest oxidative values across the genotypes. As also observed in Dalle Zotte et al. [18], chronic HS increased the meat PUFA fraction, particularly *n*-6 FA, to the detriment of total MUFAs, without affecting its oxidative status.

Neither genotype nor temperature significantly affected the sensory traits of LTL meat, including the overall perception of off-odors and off-flavors. The sole exception was the perceived meat toughness, which was more pronounced in the meat of PK rabbits, followed by the PW and PL lines, an evaluation that reflected the instrumental values.

## 5. Conclusions

As expected, the three rabbit genotypes demonstrated different performances, carcass traits and meat quality characteristics depending on their genetic background. The PL line showed better performance, with intermediate but balanced results in carcass and meat quality traits compared to the PW and PK lines. Chronic HS negatively affected all three genotypes, although to a different extent. The PW genotype exhibited the largest reduction in performance, which was probably due to a decrease in fat reserves as a result of selection to increase meat production. The PK rabbits were the most affected, especially concerning carcass and meat quality traits. Notably, the PL seemed to be the least impacted line, especially considering productive efficiency (FCR), even under chronic HS conditions. This characteristic may contribute to enhance the heat tolerance of the crossed offspring. The present results reinforce the critical role of genetic background in a line’s capacity to cope with adverse environmental factors. The results shown by the PL line suggest its potential in mitigating HS-related negative effects. However, the current paucity of scientific information regarding this particular line suggests the need for further investigation into tailored management practices, aimed at enhancing the line performance and traits under challenging environmental conditions.

## Figures and Tables

**Table 1:** Growth performance of three rabbit genotypes (G: Pannon Large, Pannon White and Pannon Ka) reared under different environmental temperatures (T: 20 °C or 28 °C).

Items	Pannon Large		Pannon White		Pannon Ka		RSD ^1^	*p*-Values		
	20 °C	28 °C	20 °C	28 °C	20 °C	28 °C		G	T	G × T
N. rabbits	60	60	60	60	60	60				
Body weight (g)										
5 weeks	874	876	848	847	843	841	2.95	<0.001	0.996	0.960
7 weeks	1754	1634	1620	1471	1541	1424	7.51	<0.001	<0.001	0.328
9 weeks	2521 ^a^	2194 ^b^	2237 ^b^	1907 ^d^	2037 ^c^	1820 ^e^	15.1	<0.001	<0.001	0.014
11 weeks	3146	2710	2779	2320	2504	2191	23.3	<0.001	<0.001	0.148
Weight gain (g/day)										
5–7 weeks	62.9	54.2	55.5	44.5	49.9	41.7	0.49	<0.001	<0.001	0.130
7–9 weeks	54.7 ^a^	41.5 ^b^	44.1 ^b^	31.2 ^d^	35.4 ^c^	28.3 ^e^	0.54	<0.001	<0.001	<0.001
9–11 weeks	47.8	39.2	38.7	29.5	33.3	26.5	0.51	<0.001	<0.001	0.382
5–11 weeks	54.4 ^a^	44.3 ^b^	46.6 ^b^	35.1 ^d^	39.6 ^c^	32.2 ^e^	0.49	<0.001	<0.001	0.046
N. cages	20	20	20	20	20	20				
Feed intake (g/day)										
5–7 weeks	124	101	119	93	120	98	1.22	<0.001	<0.001	0.167
7–9 weeks	180 ^a^	129 ^c^	155 ^b^	112 ^d^	143 ^b^	107 ^d^	2.43	<0.001	<0.001	0.001
9–11 weeks	179	134	154	111	145	103	2.47	<0.001	<0.001	0.673
5–11 weeks	161	121	143	105	138	103	1.99	<0.001	<0.001	0.283
Feed conversion ratio										
5–7 weeks	1.98	1.87	2.16	2.09	2.41	2.35	0.19	<0.001	<0.001	0.543
7–9 weeks	3.29 ^c^	3.06 ^d^	3.52 ^bc^	3.61 ^b^	4.04 ^a^	3.77 ^ab^	0.38	<0.001	0.007	0.007
9–11 weeks	3.71	3.31	4.02	3.78	4.37	3.92	0.04	<0.001	<0.001	0.317
5–11 weeks	2.91 ^c^	2.65 ^d^	3.11 ^b^	3.01 ^bc^	3.50 ^a^	3.19 ^b^	0.26	<0.001	<0.001	0.001

^1^ Residual Standard Deviation; ^a,b,c,d,e^ Values within a row with different superscripts differ significantly at *p* < 0.05.

**Table 2:** Carcass traits of three rabbit genotypes (G: Pannon Large, Pannon White and Pannon Ka) reared under different environmental temperatures (T: 20 °C or 28 °C).

Items	Pannon Large		Pannon White		Pannon Ka		RSD ^1^	*p*-Values		
	20 °C	28 °C	20 °C	28 °C	20 °C	28 °C		G	T	G × T
N. rabbits	15	15	15	15	15	15				
Slaughter Weight, SW (g)	3158 ^a^	2784 ^b^	2758 ^b^	2285 ^d^	2507 ^c^	2171 ^e^	92.6	<0.001	<0.001	0.016
Bleeding loss (% SW)	2.64	2.30	2.42	2.23	2.39	2.57	0.51	0.407	0.274	0.142
Skin (g)	473 ^a^	390 ^b^	413 ^b^	301 ^d^	362 ^c^	291 ^d^	24.7	<0.001	<0.001	0.007
Skin incidence (% SW)	14.9	13.9	14.9	13.2	14.5	13.3	0.79	0.022	<0.001	0.107
Full GIT (g)	515	451	432	365	409	373	30.9	<0.001	<0.001	0.111
GIT incidence (% SW)	16.3	16.2	15.7	15.9	16.3	17.2	1.09	0.006	0.125	0.245
Chilled Carcass, CC (g)	1906	1701	1684	1420	1525	1317	69.3	<0.001	<0.001	0.190
Carcass yield (% SW)	60.3	61.1	61.0	62.1	60.8	61.6	1.32	0.016	0.043	0.165
Carcass drip loss (%)	2.87	3.15	2.93	3.07	2.99	2.97	0.40	0.962	0.119	0.327
Liver (% CC)	4.73	4.12	4.04	3.84	4.86	4.31	0.48	<0.001	<0.001	0.213
Kidney (% CC)	0.91	0.87	0.95	0.87	0.95	0.90	0.09	0.238	0.003	0.700
Head (% CC)	7.31	7.64	7.83	8.48	8.37	8.78	0.44	<0.001	<0.001	0.351
Perirenal fat (% CC)	1.28 ^b^	0.89 ^bc^	1.02 ^abc^	0.80 ^c^	1.96 ^a^	1.25 ^b^	0.31	<0.001	<0.001	0.012
Scapular fat (% CC)	0.50	0.36	0.30	0.21	0.57	0.40	0.19	<0.001	0.002	0.699
Total dissectible fat (% CC)	1.79 ^b^	1.26 ^c^	1.33 ^b^	1.02 ^c^	2.54 ^a^	1.66 ^b^	0.43	<0.001	<0.001	0.038
Reference Carcass ^2^, RC (g)	1634	1465	1443	1213	1287	1114	61.8	<0.001	<0.001	0.109
RC yield (% CC)	85.6	86.1	85.5	85.4	84.4	84.5	0.66	<0.001	0.447	0.122
Fore part (% RC)	29.6	29.4	28.9	28.3	29.7	29.3	0.96	0.002	0.080	0.815
Mid part (% RC)	31.2	30.4	30.5	30.6	31.1	31.2	0.87	0.034	0.267	0.115
Hind part (% RC)	37.3	38.7	39.1	39.8	36.8	37.8	0.99	<0.001	<0.001	0.374
Total hind legs (% RC)	35.4	36.7	37.1	37.7	34.3	35.4	0.88	<0.001	<0.001	0.265
Total LTL muscle (% RC)	12.2	11.9	12.6	12.7	12.7	12.5	0.78	0.007	0.603	0.496
Slaughter Weight, SW (g)	3158 ^a^	2784 ^b^	2758 ^b^	2285 ^d^	2507 ^c^	2171 ^e^	92.6	<0.001	<0.001	0.016

GIT = Gastrointestinal tract; LTL = *longissimus thoracis et lumborum* muscle; ^1^ Residual Standard Deviation; ^2^ Calculated as: RC (g) = CC − (head + liver + kidney + thoracic organs + neck); ^a,b,c,d,e^ Values within a row with different superscripts differ significantly at *p* < 0.05.

**Table 3:** Physical traits of the hind leg (HL) meat of three rabbit genotypes (G: Pannon Large, Pannon White and Pannon Ka) reared under different environmental temperatures (T: 20 °C or 28 °C).

Items	Pannon Large		Pannon White		Pannon Ka		RSD ^1^	*p*-Values		
	20 °C	28 °C	20 °C	28 °C	20 °C	28 °C		G	T	G × T
N. samples	15	15	15	15	15	15				
*Biceps femoris*										
pHu	5.79	5.85	5.74	5.76	5.77	5.78	0.11	0.057	0.205	0.663
L*	51.0	51.5	50.0	50.5	48.6	50.3	2.53	0.069	0.078	0.589
a*	1.25	0.81	0.73	0.64	2.29	0.71	1.57	0.137	0.035	0.162
b*	5.48	5.53	5.66	5.71	6.36	5.35	1.49	0.660	0.345	0.291
Hind leg										
Thawing loss, %	0.31	0.31	0.60	0.50	0.48	0.68	0.31	0.002	0.600	0.165
Bones, g	36.4	35.6	33.9	30.9	31.1	28.9	2.28	0.001	0.001	0.205
Bones, % HL	12.5	13.2	12.6	13.5	14.1	14.6	0.99	0.001	0.002	0.786
Femur, g	15.7	15.2	14.4	13.9	14.1	13.5	2.37	0.034	0.296	0.990
Femur, % HL bones	42.9	42.0	42.5	45.0	45.4	46.7	6.08	0.078	0.463	0.551
Femur WBFT, N	301 ^ab^	312 ^ab^	316 ^a^	270 ^b^	305 ^ab^	285 ^ab^	37.4	0.372	0.023	0.017
Meat to bone ratio	7.01	6.60	6.89	6.42	6.09	5.83	0.56	0.001	0.002	0.789

WBFT = Warner–Bratzler fracture toughness; ^1^ Residual Standard Deviation; ^a,b^ Values within a row with different superscripts differ significantly at *p* < 0.05.

**Table 4:** Physical traits of the *longissimus thoracis et lumborum* muscle of three rabbit genotypes (G: Pannon Large, Pannon White and Pannon Ka) reared under different environmental temperatures (T: 20 °C or 28 °C).

Items	Pannon Large		Pannon White		Pannon Ka		RSD ^1^	*p*-Values		
	20 °C	28 °C	20 °C	28 °C	20 °C	28 °C		G	T	G × T
N. samples	15	15	15	15	15	15				
pHu	5.57	5.61	5.56	5.59	5.58	5.61	0.06	0.369	0.007	0.971
L*	53.2	52.2	50.8	50.3	50.9	51.8	4.65	0.202	0.688	0.882
a*	−2.93	−2.81	−2.72	−2.35	−1.89	−2.35	1.24	0.071	0.963	0.426
b*	9.86	9.26	9.41	8.74	9.13	8.59	1.30	0.109	0.030	0.981
Thawing loss, %	5.97	5.42	7.98	6.60	6.79	5.64	2.13	0.017	0.026	0.741
Cooking loss, %	20.9	22.9	22.4	22.0	23.5	25.1	3.12	0.003	0.220	0.406
Total loss, %	26.8	28.1	30.4	28.6	30.3	30.7	3.98	0.013	0.925	0.314
WBSF, N	21.8	20.0	23.6	23.0	30.6	28.3	6.54	<0.001	0.251	0.869

WBFT = Warner–Bratzler fracture toughness; ^1^ Residual Standard Deviation.

**Table 5:** Proximate composition (g/100 g meat), heme-iron, mineral content (mg/kg meat) and oxidative status (TBARs ^1^: mg MDA ^2^/kg meat) of the hind leg meat of three rabbit genotypes (G: Pannon Large, Pannon White and Pannon Ka) reared under different environmental temperatures (T: 20 °C or 28 °C).

Items	Pannon Large		Pannon White		Pannon Ka		RSD ^3^	*p*-Values		
	20 °C	28 °C	20 °C	28 °C	20 °C	28 °C		G	T	G × T
N. samples	15	15	15	15	15	15				
Water	74.6	75.7	74.6	75.2	73.7	74.8	0.51	0.001	0.001	0.054
Protein	21.6	21.5	22.0	21.8	22.0	22.0	0.32	0.001	0.087	0.717
Lipids	5.62 ^b^	4.97 ^b^	5.56 ^b^	5.00 ^b^	6.39 ^a^	5.09 ^b^	0.60	0.005	0.001	0.039
Ash	1.21	1.20	1.23	1.22	1.24	1.22	0.04	0.077	0.267	0.607
Heme iron	3.03	2.51	2.73	2.63	3.21	2.78	0.47	0.100	0.006	0.329
Ca	57.3	57.2	54.4	56.9	64.0	65.4	6.81	0.001	0.526	0.862
P	2562	2491	2531	2509	2657	2602	92.2	0.002	0.069	0.752
Na	534	574	524	521	586	576	34.4	0.001	0.365	0.095
K	4164	4138	4193	4148	4389	4378	169	0.004	0.584	0.961
TBARs	0.67	0.69	0.73	0.71	0.70	0.74	0.05	0.039	0.223	0.277

^1^ Thiobarbituric Acid Reactive substances; ^2^ Malondialdehyde; ^3^ Residual Standard Deviation; ^a,b,^ Values within a row with different superscripts differ significantly at *p* < 0.05.

**Table 6:** Fatty acid profile (% of total fatty acid methyl esters—FAMEs) of the hind leg meat of three rabbit genotypes (G: Pannon Large, Pannon White and Pannon Ka) reared under different environmental temperatures (T: 20 °C or 28 °C).

Items	Pannon Large		Pannon White		Pannon Ka		RSD ^1^	*p*-Values		
	20 °C	28 °C	20 °C	28 °C	20 °C	28 °C		G	T	G × T
N. samples	15	15	15	15	15	15				
C10:0	0.33	0.36	0.38	0.46	0.42	0.49	0.13	0.042	0.090	0.837
C12:0	0.33	0.40	0.34	0.39	0.37	0.41	0.11	0.838	0.083	0.923
C14:0	2.20	1.74	2.01	1.81	2.29	2.04	0.26	0.021	0.002	0.366
C15:0	0.58	0.68	0.62	0.72	0.55	0.70	0.05	0.025	0.001	0.278
C16:0	25.0	23.6	24.3	24.0	25.2	24.6	1.04	0.089	0.011	0.418
C17:0	0.64	0.74	0.71	0.81	0.61	0.75	0.06	0.002	0.001	0.580
C18:0	7.32	8.29	7.76	8.31	7.35	8.28	0.53	0.406	0.001	0.464
C20:0	0.22	0.20	0.24	0.19	0.21	0.18	0.03	0.123	0.002	0.627
C23:0	0.61	0.63	0.48	0.87	0.70	0.60	0.40	0.927	0.382	0.208
C24:0	0.36	0.34	0.34	0.35	0.32	0.33	0.11	0.829	0.917	0.889
C14:1	0.30	0.29	0.28	0.29	0.31	0.24	0.05	0.729	0.117	0.064
C15:1	0.22	0.21	0.21	0.21	0.20	0.22	0.08	0.958	0.838	0.859
C16:1	2.64	1.18	1.77	1.10	3.76	2.02	0.70	0.001	0.001	0.099
C17:1	0.33	0.33	0.27	0.35	0.31	0.34	0.05	0.635	0.021	0.143
C18:1 *n*-9	21.2	19.5	21.0	20.0	22.6	20.2	0.10	0.002	0.001	0.349
C18:1 *n*-11	1.29	1.26	1.24	1.20	1.39	1.20	0.12	0.257	0.014	0.121
C18:2 *n*-6	26.7	30.0	28.3	29.3	24.6	27.2	1.59	0.001	0.001	0.220
C18:3 *n*-6	0.24	0.22	0.21	0.20	0.21	0.21	0.06	0.353	0.547	0.913
C20:3 *n*-6	0.38	0.42	0.41	0.42	0.33	0.41	0.04	0.026	0.001	0.125
C20:4 *n*-6	2.53	2.85	2.45	2.71	2.27	2.74	0.36	0.364	0.002	0.692
C18:3 *n*-3	2.03	2.16	2.25	2.20	2.02	2.03	0.17	0.007	0.563	0.327
C20:5 *n*-3	0.14	0.18	0.14	0.16	0.12	0.15	0.03	0.054	0.001	0.563
C22:6 *n*-3	0.13	0.19	0.14	0.16	0.14	0.16	0.03	0.452	0.003	0.099
SFA	37.7	37.1	37.3	38.0	38.1	38.4	1.23	0.112	0.743	0.389
MUFA	26.0	22.8	24.7	22.7	28.6	24.2	1.66	0.001	0.001	0.151
PUFA	33.0	36.0	34.2	35.5	30.0	33.1	2.02	0.001	0.001	0.260
*n*-6	30.1	33.4	32.0	32.9	28.0	31.0	1.92	0.001	0.001	0.283
*n*-3	2.41	2.63	2.65	2.61	2.39	2.43	0.17	0.004	0.157	0.116
*n*-6/*n*-3	12.5	12.8	12.0	13.0	12.0	13.0	0.74	0.133	0.002	0.250
^2^ AI	0.58	0.52	0.56	0.54	0.60	0.58	0.04	0.018	0.014	0.190
^3^ TI	0.98	0.94	0.94	0.96	0.99	1.00	0.05	0.016	0.776	0.165
^4^ PI	45.1	50.2	46.9	49.0	42.0	47.0	3.02	0.001	0.001	0.322
^5^ h/H	1.95	2.15	2.06	2.10	1.88	1.97	0.13	0.005	0.005	0.199

SFA = Saturated fatty acids; MUFA = Monosaturated fatty acids; PUFA = Polyunsaturated fatty acids; ^1^ Residual Standard Deviation; ^2^ AI: Atherogenicity Index = (C12:0 + 4 × C14:0 + C16:0)/[Total MUFA + Total (*n*-6) + Total (*n*-3)]; ^3^ TI: Thrombogenicity Index = (C14:0 + C16:0 + C18:0)/[(0.5 × Total MUFA) + 0.5 × (*n*-6) + 3 × (*n*-3)/(*n*-6)]; ^4^ PI: Peroxidability Index = (% monoenoic × 0.025) + (% dienoic × 1) + (% trienoic × 2) + (% tetraenoic × 4) + (%pentaenoic × 6) + (% hexaenoic × 8); ^5^ h/H: hypocholesterolemic/Hypercholesterolemic Index = [(C18:1 *n*-9 + C18:2 *n*-6 + C20:4 *n*-6 + C18:3 *n*-3 + C20:5 *n*-3 + C22:5 *n*-3 + C22:6 *n*-3)/(C14:0 + C16:0).

**Table 7:** Sensory score of the *longissimus thoracis et lumborum* meat of three rabbit genotypes (G: Pannon Large, Pannon White and Pannon Ka) reared under different environmental temperatures (T: 20 °C or 28 °C).

Items	Pannon Large		Pannon White		Pannon Ka		RSD ^1^	*p*-Values		
	20 °C	28 °C	20 °C	28 °C	20 °C	28 °C		G	T	G × T
N. samples	15	15	15	15	15	15				
Rabbit odor intensity	4.95	4.17	4.29	4.83	4.94	4.55	2.27	0.934	0.657	0.515
Overall off-odors intensity	1.37	0.99	0.73	1.04	0.88	0.73	1.01	0.371	0.755	0.450
Liver odor	1.52	0.79	1.08	1.09	1.42	0.19	1.53	0.722	0.083	0.368
Rabbit flavor intensity	5.01	4.88	4.89	5.08	4.79	5.31	2.25	0.984	0.696	0.858
Overall off-flavor intensity	1.44	1.45	1.70	2.00	1.71	1.21	1.88	0.674	0.883	0.730
Liver flavor	1.13	0.95	1.61	0.48	0.87	0.91	1.54	0.936	0.263	0.398
Toughness	4.81	5.31	5.49	4.36	6.85	6.32	2.66	0.032	0.492	0.490
Juiciness	6.07	6.72	6.39	6.63	5.28	6.11	2.94	0.510	0.364	0.926
Fibrousness	6.93	6.97	6.57	5.43	7.09	6.65	2.76	0.341	0.382	0.705
Greasiness	3.30	2.63	3.90	3.58	4.26	3.11	2.43	0.396	0.176	0.813

^1^ Residual Standard Deviation.

**Table 8:** Off-odor and off-flavor perception in the *longissimus thoracis et lumborum* meat of three rabbit genotypes (Pannon Large, Pannon White and Pannon Ka) reared under different environmental temperatures (20 °C or 28 °C).

Items	Pannon Large		Pannon White		Pannon Ka		*p*-Value	χ^2^
	20 °C	28 °C	20 °C	28 °C	20 °C	28 °C		
N. samples	15	15	15	15	15	15		
Off-odors:								
Pungent	0.00	6.70	0.00	13.3	0.00	6.70	0.388	5.23
Cardboard	20.0	20.0	33.3	26.7	6.70	40.0	0.354	5.54
Livery	33.3	13.3	26.7	13.3	26.7	13.3	0.618	3.54
Rancid	0.00	6.70	6.70	0.00	0.00	0.00	0.536	4.09
Off-flavors:								
Pungent	0.00	0.00	0.00	6.70	13.3	6.70	0.388	5.23
Cardboard	13.3	13.3	13.3	13.3	13.3	20.0	0.994	0.45
Acid	6.70	6.70	6.70	0.00	13.3	0.00	0.608	3.60
Metallic	26.7	20.0	26.7	6.70	13.3	13.3	0.647	3.35
Livery	20.00	26.7	26.7	20.0	20.0	6.70	0.776	2.50
Rancid	0.00	6.70	13.3	6.70	0.00	6.70	0.608	3.60

## Data Availability

The raw data supporting the conclusions of this article will be made available by the authors upon request.
